# Carbon isotope discrimination as a diagnostic tool for C_4_ photosynthesis in C_3_-C_4_ intermediate species

**DOI:** 10.1093/jxb/erv555

**Published:** 2016-02-08

**Authors:** Hugo Alonso-Cantabrana, Susanne von Caemmerer

**Affiliations:** Division of Plant Sciences, Research School of Biology, The Australian National University, Canberra, ACT 0200, Australia

**Keywords:** Carbon isotope discrimination, C_3_-C_4_, intermediate photosynthesis, *Flaveria*, *F. brownii*, *F. floridana*.

## Abstract

The presence and activity of the C_4_ cycle in C_3_-C_4_ intermediate species have proven difficult to analyze, especially when such activity is low. This study proposes a strategy to detect C_4_ activity and estimate its contribution to overall photosynthesis in intermediate plants, by using tunable diode laser absorption spectroscopy (TDLAS) coupled to gas exchange systems to simultaneously measure the CO_2_ responses of CO_2_ assimilation (*A*) and carbon isotope discrimination (*Δ*) under low O_2_ partial pressure. Mathematical models of C_3_-C_4_ photosynthesis and *Δ* are then fitted concurrently to both responses using the same set of constants. This strategy was applied to the intermediate species *Flaveria floridana* and *F. brownii*, and to *F. pringlei* and *F. bidentis* as C_3_ and C_4_ controls, respectively. Our results support the presence of a functional C_4_ cycle in *F. floridana*, that can fix 12–21% of carbon. In *F. brownii*, 75–100% of carbon is fixed via the C_4_ cycle, and the contribution of mesophyll Rubisco to overall carbon assimilation increases with CO_2_ partial pressure in both intermediate plants. Combined gas exchange and *Δ* measurement and modeling is a powerful diagnostic tool for C_4_ photosynthesis.

## Highlight

Combined gas exchange and carbon isotope discrimination measurement and modeling is used to detect and estimate the contribution of the C_4_ cycle in the C_3_-C_4_ intermediate species.

## Introduction

C_4_ photosynthesis is a highly efficient carbon fixation system characterized by the presence of a biochemical carbon pump with the capacity of increasing the CO_2_ partial pressure (*p*CO_2_) at the site of ribulose 1,5-bisphosphate carboxylase/oxygenase (Rubisco) to concentrations higher than ambient air (Hatch *et al.*, 1967; Hatch, 1987; Ehleringer *et al.*, 1991). This increases photosynthetic rates and reduces photorespiration, potentially improving nitrogen and water use efficiency (Hibberd *et al.*, 2008; Langdale, 2011). Most C_4_ species show a common anatomical pattern, called Kranz anatomy, that leads to the separation of enzyme functions in two compartments, the mesophyll and the bundle sheath cell (Brown, 1975). CO_2_ is first hydrated into bicarbonate in the mesophyll cell cytoplasm in a reversible reaction catalyzed by carbonic anhydrase (CA) (Badger and Price, 1994). Carbon is then fixed by phosphoenol pyruvate carboxylase (PEPC), localized exclusively in the mesophyll, into four-carbon acids that diffuse to the internally adjacent bundle sheath cell, where they are decarboxylated and the released CO_2_ is refixed by Rubisco.

The most productive crops, such as maize, sorghum and sugar cane, are C_4_ plants, exemplifying the higher efficiency of this system over the C_3_ photosynthetic pathway present in most plant species, including major crops like wheat and rice. For this reason, there is currently a strong interest in implementing the advantages of C_4_ photosynthesis in to C_3_ crops with the aim of increasing yield, to keep pace with the food needs of a growing world population (von Caemmerer *et al.*, 2012; Karki *et al.*, 2013; Leegood, 2013). This kind of approach is boosting research on genetic, biochemical and physiological aspects of C_4_ photosynthesis. However, the initial phases of these initiatives are not expected to produce fully functional C_4_ plants, but plants showing incomplete C_4_ phenotypes like those observed in C_3_-C_4_ intermediate species, which have been considered remnants of the evolution from C_3_ ancestors to C_4_ plants (Rawsthorne, 1992; Sage *et al.*, 2011). They show Kranz or Kranz-like leaf anatomy, but the activity of C_4_-related enzymes, such as PEPC, is lower compared to strict C_4_ plants, and enzyme compartmentation is incomplete, with Rubisco and PEPC present in both the mesophyll and the bundle sheath cells (Cheng *et al.*, 1988; Brown and Hattersley, 1989; Byrd *et al.*, 1992). These factors reduce the efficiency of the carbon concentrating mechanism. In intermediate plants, a photorespiratory CO_2_ pump, also known as the C_2_ cycle or glycine shuttle, transports glycine formed during mesophyll photorespiration to the bundle sheath where it is decarboxylated and the CO_2_ refixed, thus increasing overall CO_2_ assimilation rate and reducing the effect of photorespiration (Monson *et al.*, 1984; Sage *et al.*, 2012; Schulze *et al.*, 2013; Keerberg *et al.*, 2014). The genus *Flaveria* has been the focus of numerous studies in the past because it comprises C_3_, C_4_ and C_3_-C_4_ intermediate species, the later showing different degrees of C_4_ activity (Ku *et al.*, 1983; McKown *et al.*, 2005).

The C_4_ cycle contribution to growth has been difficult to quantify in intermediate species. In these plants, a steeper initial slope in the CO_2_ response of the CO_2_ assimilation rate compared to a strict C_3_ plantis expected. However, this trait is also affected by Rubisco content and its kinetic properties, so conclusions are not straightforward (von Caemmerer, 2000; von Caemmerer and Quick, 2000). Another important manifestation of C_4_ activity in intermediate species is a reduction of the O_2_ sensitivity of CO_2_ assimilation and the compensation point (Γ) due to a proportion of Rubisco being contained in the bundle sheath (BS) and thus not in direct contact with air (Byrd and Brown, 1989; Dai *et al.*, 1996). With the photorespiratory pump causing a similar effect, separating and quantifying the contribution of each biochemical pathway through this approach is not possible. The C_4_ cycle activity relative to overall photosynthesis in intermediates has been estimated in the past by metabolite profiling, but recent reports indicate that metabolite accumulation is strongly dependent on the leaf zone sampled and its developmental stage (Monson *et al.*, 1986; Leegood and von Caemmerer, 1994; Wang *et al.*, 2014).

In order to develop a deeper understanding of the physiology of both natural and artificial C_3_-C_4_ intermediates, better tools are needed to evaluate the contribution of C_4_ photosynthesis to overall assimilation. One signature of the activity of PEPC as the initial CO_2_ fixation enzyme is a change in carbon isotopic discrimination (*Δ*) during photosynthesis. Whereas Rubisco has a strong preference for the lighter isotope, ^12^C, over the heavier isotope, ^13^C, PEPC is less discriminating, which causes an important difference in the biochemical fractionation between C_3_ and C_4_ plants (O’Leary, 1981; Farquhar, 1983). Incomplete C_4_ photosynthesis in C_3_-C_4_ intermediates is also reflected in *Δ*, with both PEPC and mesophyll Rubisco acting as the initial CO_2_ fixing enzymes and their relative activities determining the resulting *Δ*. Mathematical models describing CO_2_ assimilation and isotopic discrimination in these plants have been previously developed (von Caemmerer and Hubick, 1989; von Caemmerer, 1992). However, attempts to characterize *Flaveria* intermediate species by studying carbon-isotope ratios in dry matter resulted in C_3_-like profiles, and were interpreted as having little or no contribution of the C_4_ system to plant growth, which was in contradiction to results from metabolite analysis (Monson *et al.*, 1988; Byrd *et al.*, 1992).

Tunable diode laser (TDL) absorption spectroscopy allows relatively rapid measurements of *Δ* concurrently with gas exchange, and has been used to analyze and compare C_3_ and C_4_ species (Tazoe *et al.*, 2011; von Caemmerer *et al.*, 2014). The present work uses this technique, combined with mathematical modeling, as a tool to determine the presence and contribution of C_4_ photosynthesis in C_3_-C_4_ intermediate plants. An updated mathematical model of carbon isotope discrimination for C_3_-C_4_ intermediate species is proposed, which considers the effect of mesophyll conductance and allows the calculation of the biochemical fractionation. The strategy was applied to the study of *Flaveria bidentis* (C_4_), *F. pringlei* (C_3_), *F. floridana* (C_3_-C_4_) and *F. brownii* (C_4_-like). *F. floridana* has been described as a C_2_ plant with elevated PEPC activity, but it was unclear if a C_4_ cycle is actually contributing to total carbon assimilation in this species (Monson *et al.*, 1986, 1988; Leegood and von Caemmerer, 1994; Dai *et al.*, 1996). *F. brownii*, on the other hand, was initially considered a C_4_ species, but later experiments proved incomplete enzyme compartmentation, with a small proportion of Rubisco activity present in the mesophyll cells, and it was then reclassified as a C_4_-like intermediate species (Holaday *et al.*, 1984; Monson *et al.*, 1987; Moore *et al.*, 1989). In the present study, concurrent *Δ* and gas exchange measurement and modeling allowed the detection and estimation of the C_4_ cycle in the intermediate species, proving itself as a powerful diagnostic tool for C_4_ photosynthesis.

## Materials and methods

### Plant material and growth conditions

*Flaveria bidentis* was propagated from seeds and *F. pringlei*, *F. brownii* and *F. floridana* were propagated from cuttings (Brown and Hattersley, 1989; Whitney *et al.*, 2011). Plants were grown in 30 l pots in a garden soil mix fertilized with Osmocote (Scotts, Australia) in a glasshouse under natural light conditions, at 28/18°C day/night temperatures, respectively. Pots were watered daily.

### Responses of CO_2_ assimilation rate and CO_2_ compensation point to O_2_ partial pressure

Two Li-Cor 6400XTs (Li-Cor, USA) were used to measure CO_2_ assimilation at a range of reference *p*CO_2_ (388, 0, 24, 48, 73, 97, 145, 194, 291, 388, 485, 582 and 776 μbar). N_2_ and O_2_ were mixed in different ratios by mass flow controllers (Omega Engineering Inc., USA) to generate a range of O_2_ partial pressures (*p*O_2_; 20, 50, 100, 200 and 300mbar) supplied to the LI-6400s. Response curves of CO_2_ assimilation rate (*A*) to intercellular *p*CO_2_ (C_*i*_), *A*/*C*_i_ curves, were repeated sequentially at each *p*O_2_. The measurements were made at 25°C, a flow rate of 500 μmol s^−1^ and 1500 μmol quanta m^−2^ s^−1^, inside a growth cabinet at 25°C. Four plants from each species were analyzed. The compensation point (Γ) was calculated from the *A*/*C*_i_ curves at each *p*O_2_, as the intercellular CO_2_ concentration where net CO_2_ assimilation is zero.

To study the inhibitory effect of O_2_ on assimilation rate, we compared the CO_2_ assimilation rate at a reference *p*CO_2_ of 380 μbar at each *p*O_2_.

### Concurrent gas exchange and *Δ* measurements and calculations of mesophyll conductance

Two Li-Cor 6400XTs (Li-Cor, USA) coupled to a tunable-diode laser absorption spectroscope (TDLAS, model TGA100A, Campbell Scientific, Inc., USA) as described in Tazoe *et al.* (2011) were used for concurrent measurements of gas exchange and carbon isotope discrimination (Bowling *et al.*, 2003; Griffis *et al.*, 2004; Pengelly *et al.*, 2012; Evans and von Caemmerer, 2013). Plants were transferred from the glasshouse to a growth cabinet with fluorescence lights (TRIL1175, Thermoline Scientific Equipment, Australia) at 25°C and one young fully expanded leaf was placed in each of the 6cm^2^ leaf chambers. Measurements were made at a leaf temperature of 25°C, a flow rate of 200 μmol s^−1^, 1500 μmol quanta m^−2^ s^−1^ and 20mbar *p*O_2_. The desired *p*O_2_ was achieved as described above and supplied to the Li-Cors 6400. Reference *p*CO_2_ was changed stepwise to 392, 980, 686, 490, 294, 196, 98, 49 and 392 μbar and measurements were made every 4min for at least 30min at each *p*CO_2_. Dark respiration (*R*_d_) was measured at the end of an *A*/*C*_i_ curve at 392 μbar *p*CO_2_ and 20mbar *p*O_2_ by switching off the Li-Cor lamp. Three or four plants from each species were analyzed. *Δ* was calculated as previously described (Evans *et al.*, 1986; Evans and von Caemmerer, 2013).

Mesophyll conductance (*g*_m_) was calculated for *F. pringlei* from concurrent gas exchange and *Δ* measurements at the above range of reference *p*CO_2_ and 19mbar *p*O_2_, applying the equations previously described and including the ternary effects of transpiration rate (Farquhar and Cernusak, 2012; Evans and von Caemmerer, 2013). This method is only valid for C_3_ species. For intermediate and C_4_ species, we assumed the same CO_2_ response of *g*_m_ found in *F. pringlei*, and scaled the absolute value at ambient *p*CO_2_ to obtain the best fit of the *A* and *Δ* models for the observed results (see Results section).

### Mathematical models

The overall rate of net CO_2_ assimilation (*A*) for C_3_-C_4_ intermediate plants was previously described (von Caemmerer, 1992, 2013):

$$A=\;A_{\text{s}}+\;A_{\text{m}}$$ (1)

where *A*_m_ is the assimilation in the mesophyll and *A*_s_ is the assimilation in the bundle sheath, which are defined as:

$$A_{\text{s}}=V_{\text{p}}+\beta F_{\text{m}}-L$$ (2)

$$A_{\text{m}}=V_{\text{m}}-R_{\text{m}}-\left(1-\beta \right)F_{\text{m}}$$ (3)

so:

$$A=V_{\text{m}}-R_{\text{m}}-F_{\text{m}}+V_{\text{p}}-L$$ (4)

where *V*_p_ is PEPC carboxylation and *β* is the fraction of the CO_2_ produced from photorespiration in the mesophyll (*F*_m_) that is released in the bundle sheath. For simplification, bundle sheath respiration and photorespiration are not taken into account in eq. 4. The term *L* is the leak rate of CO_2_ out of the bundle sheath, and can be expressed as:

$$L=\phi (V_{\text{p}}+\beta F_{\text{m}})$$ (5)

and

$$A=V_{\text{m}}-R_{\text{m}}-F_{\text{m}}+V_{\text{p}}-\phi (V_{\text{p}}+\beta F_{\text{m}})$$ (6)

where *ϕ* (leakiness) is the ratio of the leak rate of CO_2_ out of the bundle sheath and the supply rate of CO_2_ to the bundle sheath $\left(V_{\text{p}}+\beta F_{\text{m}}\right)$ . When *p*O_2_ is low, *F*_m_ can be considered 0.

*V*_m_ and *R*_m_ are Rubisco carboxylation and day respiration in the mesophyll, respectively. *V*_p_ and *V*_m_ are calculated as described in von Caemmerer (2000):

$$V_{\text{m}}=\frac{C_{\text{m}}\cdot V_{\text{m},\text{max}}}{C_{\text{m}}+K_{\text{c}}(1+\frac{\text{O}}{K_{\text{o}}})}$$ (7)

$$V_{\text{p}}=\frac{C_{\text{m}}\cdot V_{\text{p},\text{max}}}{C_{\text{m}}+K_{\text{p}}}$$ (8)

and

$$C_{\text{m}}=C_{\text{i}}-\frac{A}{g_{\text{m}}}$$ (9)

where *C*_m_ and *C*_i_ are mesophyll and intercellular *p*CO_2_, respectively. *K*_c_ and *K*_o_ are the Michaelis-Menten constants for CO_2_ and O_2_ respectively, expressed as a partial pressure. Although the *p*CO_2_ in the cytosol (site of PEPC carboxylation) and the chloroplast (site of Rubisco carboxylation) of the mesophyll cell are presumably different due to diffusional limitations, the same value (*C*_m_) was assumed in both compartments (von Caemmerer, 2000, 2013; Tholen and Zhu, 2011).

When the rate of PEP regeneration is limiting, *V*_p_=*V*_pr_, where *V*_pr_ is a constant. *V*_m,max_ is the maximum Rubisco carboxylation in the mesophyll, and *V*_p,max_ is the maximum PEPC carboxylation (Table 1). When RuBP becomes limiting, *V*_m_ in eq. 6 can be given by an electron transport limited rate (*W*_j_), as previously described (von Caemmerer, 2000, 2013).

**Table 1.** Values assigned to variables for model fitting purposes When fitting *F. brownii* as a strict C_4_ and *F. floridana* as a strict C_3_ species, values were assigned to obtain the best fitting without considering measured enzyme activities.

**Variable**	**Definition**	***F. pringlei***	***F. bidentis***	***F. brownii***	***F. brownii* (strict C**_**4**_)	***F. floridana***	***F. floridana* (strict C**_**3**_)	**Origin of the value**
a	Fractionation during diffusion in air (‰)	4.4	4.4	4.4	4.4	4.4	4.4	Farquhar (1983)
a_b_	Fractionation during diffusion through the boundary layer (‰)	2.9	2.9	2.9	2.9	2.9	2.9	Griffiths *et al.* (2007)
abs	Leaf absorptance	0.8	0.8	0.8	0.8	0.8	0.8	von Caemmerer (2000)
a_l_	Fractionation during diffusion in water (‰	0.7	0.7	0.7	0.7	0.7	0.7	Griffiths *et al.* (2007)
β	Fraction of the photorespired CO_2_ released in the bundle sheath	1	1	1	1	1	1	Assigned
b_3_	Fractionation during carboxylation by Rubisco (‰)	29	29	29	29	29	29	Roeske and O’Leary (1984)
b_4_	Combined fractionation by the C_4_ cycle (‰)	na	−5.7	−5.7	−5.7	−5.7	na	O’Leary (1981)
b_s_	Fractionation during CO_2_ dissolution in water (‰	1.1	1.1	1.1	1.1	1.1	1.1	von Caemmerer (1992)
c	g_m_ scaling constant	0.666^a^	0.8^b^	0.666^b^	0.666^b^	0.78^b^	0.78^b^	^a^, measured in this work; ^b^, assigned from model fitting
e	fractionation during mitochondrial respiration	2.91	3.54	3.51	3.51	3.72	3.72	Calculated as e=δ^13^C_cylinder_-δ^13^C_atmosphere_
F	Correction coefficient for spectral quality	0.15	0.15	0.15	0.15	0.15	0.15	von Caemmerer (2000)
J_t_	Total electron transport rate (μmol electrons m^−2^ s^−1^)	120	400	440	700	250	0	Assigned [von Caemmerer (2000), eq. 5.17]
J_m_	Electron transport rate allocated to mesophyll C_3_ cycle	120	0	40	0	200	240	Assigned [von Caemmerer (2000), eq. 5.17]
K_C_	Rubisco Michaelis–Menten constant for CO_2_ (μbar)	359	605	383	383	395	395	Kubien *et al.* (2008)
K_O_	Rubisco Michaelis–Menten constant for O_2_ (μbar)	528 000	507 000	300 000	300 000	544 000	544 000	Kubien *et al.* (2008)
K_P_	PEPC Michaelis–Menten constant for PEP (μbar)	n.a.	80	80	n.a.	80	n.a.	Bauwe (1986)
R_d_	Mitochondrial respiration (μmol m^−2^ s^−1^)	0.6	0.4	1.3	1.3	1.7	1.7	Measured in the dark in this work
s	Fractionation during leakage (‰)	n.a.	1.8	1.8	1.8	1.8	n.a.	von Caemmerer (1992)
V_m, max_	Maximum Rubisco carboxylation rate in the mesophyll (μmol m^−2^ s^−1^)	60^a^	0^b^	15^b^	0^b^	90^a^	130^b^	^a^, measured in this work; ^b^, assigned
V_P, max_	Maximum PEP carboxylation rate (μmol m^−2^ s^−1^)	0^a^	90^a^	80^a^	80^b^	15^a^	0^b^	^a^, measured in this work; ^b^, assigned
V_Pr_	PEP regeneration rate (μmol m^−2^ s^−1^)	0	36	32	50	8	0	Assigned
*φ*	Leakiness	n.a.	0.28	0.21	0.3	0.40	n.a.	Assigned from model fitting
θ	Empirical curvature factor	0.1	0.1	0.1	0.1	0.1	0.1	Ubierna *et al.* (2011)

n.a., not applicable.

Theory developed by Farquhar *et al.* (1982) and Farquhar (1983) showed that photosynthetic carbon isotope discrimination can be described by equations having diffusion and biochemistry dependent terms. The equation of *Δ* presented by (Griffiths *et al.*, 2007), which takes into account the effect of *g*_m_, was modified to incorporate the ternary effects of transpiration rate as suggested by Farquhar and Cernusak (2012):

$$\begin{array}{c} \Delta =\frac{1}{1-t}a^{\prime }+\frac{1+t}{1-t}(a_{\text{l}}+b_{\text{s}}-\Delta_{\text{bio}})\frac{A}{g_{\text{m}}\cdot C_{\text{a}}}\\ +\frac{1}{1-t}[\left(1+t\right)\Delta_{bio}-a^{\prime }]\frac{C_{\text{i}}}{C_{\text{a}}} \end{array}$$ (10)

where *a*_l_ is the fractionations during diffusion in water and *b*_*s*_ is the fractionation as CO_2_ enters solution. The term $t=\frac{\left(1+a^{'}\right)E}{2g_{ac}^{t}}$ , where *E* denotes the transpiration rate and $g_{ac}^{t}$ the total conductance to CO_2_ diffusion including boundary layer and stomatal conductance. The symbol *aʹ* denotes the combined fractionation during diffusion in the boundary layer and in air, and is calculated as:

$$a^{\prime }=\frac{a_{\text{b}}\left(C_{\text{a}}-C_{\text{l}}\right)+a(C_{\text{l}}-C_{\text{i}})}{\left(C_{\text{a}}-C_{\text{i}}\right)}$$ (11)

where *a* is the fractionation during diffusion in air, *a*_b_ is the fractionation during diffusion in the boundary layer, and *C*_a_, *C*_l_, *C*_i_ are the *p*CO_2_ in the air, leaf surface and intercellular space respectively. The biochemical fractionation, *Δ*_bio_, is the integrated net biochemical discrimination, and depends on the biochemistry of net CO_2_ uptake (Griffiths *et al.*, 2007).

When *Δ* and *g*_m_ are known, *Δ*_bio_ can be solved from equation 10, resulting in:

$$\Delta_{\text{bio}}=\frac{\Delta -\frac{1}{1-t}a'\frac{C_{a}-C_{i}}{C_{a}}-\frac{1+t}{1-t}a_{i}\frac{A}{g_{\text{m}}\cdot C_{\text{a}}}}{\frac{1+t}{1-t}(\frac{C_{\text{i}}}{C_{\text{a}}}-\frac{A}{g_{\text{m}}\cdot C_{\text{a}}})}$$ (12)

Because *g*_m_ was obtained from combined measurement of *Δ* and gas exchange in the C_3_ species *F. pringlei*, *Δ* and *g*_m_ are not independent and we could not estimate *Δ*_bio_ from eq. 12. For the intermediate and C_4_ species, *g*_m_ was calculated independently of the *Δ* measurements as described in the Materials and Methods section, so *Δ*_bio_ could be estimated from eq. 12 for *F. floridana*, *F. brownii* and *F. bidentis*.

For modeling purposes, or when *Δ* is unknown, *Δ*_bio_ can be derived from von Caemmerer’s (1992) equation A17:

$$\begin{array}{l} \frac{R_{\text{i}}}{R_{\text{p}}}=1+(b_{3}-\frac{fF_{\text{m}}+eR_{\text{m}}}{A})+\frac{A_{\text{s}}}{A}[\left(b_{3}-s\right)\phi \\ \;+\frac{\left(b_{4}-b_{3}\right)V_{\text{p}}-f\beta F_{\text{m}}}{V_{\text{p}}+\beta F_{\text{m}}}]-\frac{fF_{\text{s}}+eR_{\text{s}}}{A}\phi \end{array}$$

where *R*_i_ and *R*_p_ are the molar abundance ratios of ^13^C/^12^C in the intercellular space and the photosynthetic product, respectively.

$$\Delta_{\text{bio}}=\frac{R_{\text{i}}}{R_{\text{p}}}-1$$

Thus:

$$\begin{array}{l} \Delta_{\text{bio}}=(b_{3}-\frac{fF_{\text{m}}+eR_{\text{m}}}{A})+\frac{A_{\text{s}}}{A}[\left(b_{3}-s\right)\phi \\ \;+\;\frac{\left(b_{4}-b_{3}\right)V_{\text{p}}-f\beta F_{\text{m}}}{V_{\text{p}}+\beta F_{\text{m}}}]-\frac{fF_{\text{s}}+eR_{\text{s}}}{A}\phi \end{array}$$ (13)

The factor *b*_*3*_ is the Rubisco fractionation, and *b*_*4*_ is the combined fractionation of PEP carboxylation and the preceding isotope equilibrium during dissolution of CO_2_ and conversion to bicarbonate; *s* is the fractionation during leakage of CO_2_ out of the bundle sheath; *e* is the fractionation during mitochondrial respiration; *f* is the fractionation during photorespiration; *R*_m_ and *R*_s_ are the mitochondrial respiration rates in the mesophyll and the bundle sheath in the light, respectively. It was assumed that *R*_d_=*R*_m_+*R*_s_, and *R*_m_=*R*_s_=0.5*R*_d_. The factors *F*_m_ and *F*_s_ are the photorespiration rates derived from Rubisco oxygenation in the mesophyll and the bundle sheath, respectively. When *p*O_2_ is low, *F*_m_ and *F*_s_ are close to 0, so equation 13 simplifies to:

$$\Delta_{\text{bio}}=(b_{3}-\frac{eR_{\text{m}}}{A})+\frac{A_{\text{s}}}{A}[\left(b_{3}-s\right)\phi +\left(b_{4}-b_{3}\right)]-\frac{eR_{\text{s}}}{A}\phi$$ (14)

The parameter *e* needs to account for differences between the isotopic composition of CO_2_ during plant growth and during the measurements, because the substrates used during respiration are most likely carbohydrates assimilated before the experiment (Wingate *et al.*, 2007). No fractionation during mitochondrial respiration was assumed in this work, so *e* was calculated as the difference between δ^13^C in the CO_2_ cylinder used during the experiments and δ^13^C in the atmosphere during growth conditions (*e*=δ^13^C_cylinder_-δ^13^C_atmosphere_) (Tazoe *et al.*, 2009; Pengelly *et al.*, 2010). In this work, δ^13^C_cylinder_ was between −4.12‰ and −5.14‰, and δ^13^C_atmosphere_ was assumed to be −8‰ (Table 1).

### *In vitro* enzyme activity assays

Leaf discs (0.5cm^2^) were collected from the leaves used for gas exchange experiments and frozen in liquid nitrogen immediately after the experiment. Soluble protein was extracted by grinding one frozen leaf disc in a cold Tenbroeck homogenizer with 0.5ml extraction buffer [50mM HEPES, 1mM EDTA, 0.1% (v/v) Triton X-100, 10mM DTT, 1% (w/v) PVPP, 1% (v/v) protease inhibitor cocktail (Sigma), pH 7.8]. Extracts were centrifuged at 13000rpm for 30s. Spectrophotometric assays were performed to determine Rubisco and PEPC activities as described in Pengelly *et al.* (2010).

CA activity was measured in the same extract used for PEPC and Rubisco activity measurements, using a membrane inlet mass spectrometer to measure the rates of ^18^O exchange from labeled ^13^C^18^O_2_ to H_2_^16^O at 25°C with a subsaturating total carbon concentration of 1mM (Badger and Price, 1989; von Caemmerer *et al.*, 2004; Cousins *et al.*, 2008). The hydration rates were calculated from the enhancement in the rate of ^18^O loss over the uncatalyzed rate, and the nonenzymatic first-order rate constant was applied at pH 7.4 (*k*_c_=6.22x10^−11^/[H^+^]+3.8x10^−2^=0.0396), appropriate for the mesophyll cytosol, at a CO_2_ concentration of 8μM, which is approximately the CO_2_ concentration in the mesophyll of *F. bidentis* (Jenkins *et al.*, 1989; von Caemmerer *et al.*, 2004). When CA is in the chloroplast, which is tipically the case in C_3_ plants like *F. pringlei*, our calculations underestimate its *in planta* activity by ~10% due to the effect of the higher chloroplastic pH on *k*_c_ (*k*_c_=0.0442 at pH 8).

## Results

### O_2_ response of CO_2_ assimilation rate and compensation point

**Fig. 1.**
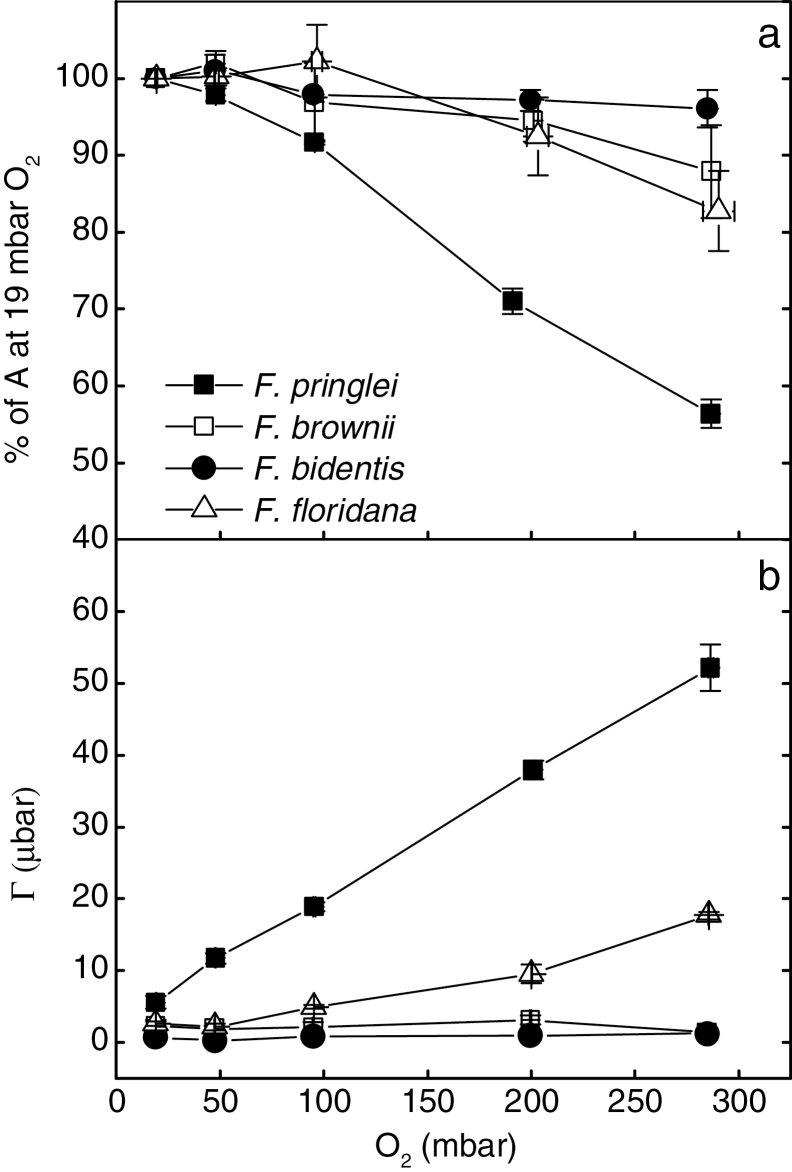
The responses of (a) CO_2_ assimilation rate, *A* and (b) compensation point (*Γ*) in *F. pringlei*, *F. floridana*, *F. brownii* and *F. bidentis* to changes in atmospheric *p*O_2_. Assimilation rate is expressed as a percentage of the assimilation rate at 19mbar O_2_ (average of 28.7±1.13 μmol m^−2^ s^−1^ for *F. pringlei*, 24.2±1.52 for *F. floridana*, 20.6±1.2 for *F. brownii* and 21.7±0.49 for *F. bidentis*). Measurements were made at 25°C and 385 μbar CO_2_ (R), and an irradiance of 1500 μmol m^−2^ s^−1^. Values represent averages and standard error of four replicates.

The effect of *p*O_2_ on CO_2_ assimilation rate and the compensation point (Γ) was measured at 380 μbar reference CO_2_, an irradiance of 1500 μmol quanta m^−2^ s^−1^ and 25 °C (Fig. 1).

In *F. pringlei*, increasing *p*O_2_ caused a decrease in CO_2_ assimilation rate, a response typical of a C_3_ plant. Consistent with this, the Γ increased with increasing *p*O_2_, ranging from 5.6 ubar at 19mbar O_2_ to 53 µbar at 285mbar O_2_.

In the C_4_ species *F. bidentis*, the effect of oxygen was very small, with only a 5% decrease in CO_2_ assimilation rate at the highest tested *p*O_2_. Γ in these plants barely changed with *p*O_2_, and ranged from 0.2 to 1.2 μbar.

The effect of O_2_ on Γ in *F. brownii* was also very small and similar to the C_4_ species *F. bidentis,* ranging from 1.3 to 3.1 μbar (Fig. 1b). However, the inhibitory effect of O_2_ on CO_2_ assimilation rate was more pronounced, and resulted in an intermediate response of CO_2_ assimilation rate to increasing *p*O_2_ (Fig. 1a).

The O_2_ response of Γ in *F. floridana* was intermediate between C_3_ and C_4_ species (2.3–18μbar; Fig. 1b), as has been previously shown (Ku *et al.*, 1991). However, in our experiments the inhibitory effect of O_2_ on photosynthesis was smaller than that previously reported by these authors and strikingly similar to that in *F. brownii* when *p*O_2_ was 200mbar or lower, despite the important differences in the enzyme compartmentation between these two species (Fig. 1a). Only at 290mbar O_2_ the inhibition of photosynthesis was higher for *F. floridana*, with a reduction of a 22%, compared to that in *F. brownii* (15% inhibition).

Stomatal conductance and *C*_i_ increased slightly with *p*O_2_, with the exception of *F. bidentis*, which remained stable, and were considerably higher in the C_3_ species *F. pringlei* at any *p*O_2_ (Supplementary Fig. S1 at *JXB* online).

### Rubisco, PEPC and CA activity

**Fig. 2.**
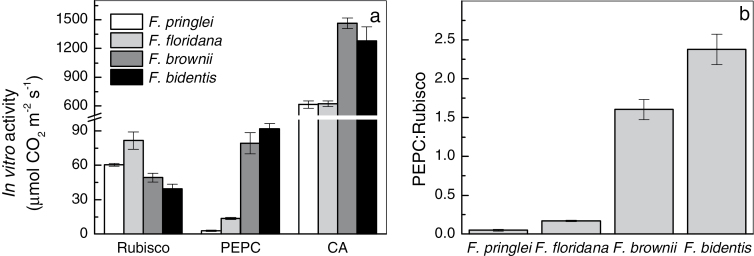
(a) *In vitro* Rubisco, PEPC and CA activities in *F. pringlei*, *F. floridana*, *F. brownii* and *F. bidentis*, measured from samples of the same leaves used for gas exchange and expressed on a leaf area basis. (b) PEPC to Rubisco activity ratio in these experiments. Values represent mean and standard error of four experimental replicates.

*In vitro* Rubisco, PEPC and CA activities were analyzed in extracts from the same leaves on which the concurrent gas exchange and *Δ* measurements were made (Fig. 2). Rubisco activity was higher in *F. floridana* (average of 74.9 μmol m^−2^ s^−1^), followed by *F. pringlei* (60.5 μmol m^−2^ s^−1^), *F. brownii* (49.2 μmol m^−2^ s^−1^) and *F. bidentis* (39.7 μmol m^−2^ s^−1^). PEPC activity was lowest in *F. pringlei* (2.9 μmol m^−2^ s^−1^) and, notably, four times higher in *F. floridana* (13.8 μmol m^−2^ s^−1^). *F. brownii* showed a PEPC activity closer to that of *F. bidentis* (79.3 and 91.8 μmol m^−2^ s^−1^ respectively). CA activity was similar and high in *F. bidentis* and *F. brownii* (1278.7 and 1464.5 μmol m^−2^ s^−1^ respectively), and lower in *F. pringlei* and *F. floridana* (614.9 and 623.6 μmol m^−2^ s^−1^ respectively).

The relative activity of PEPC to Rubisco was lowest in *F. pringlei* and highest in *F. bidentis* (Fig. 2b). *F. floridana* showed a PEPC:Rubisco ratio 3.4 times greater than the C_3_ species, and *F. brownii* was closer to the C_4_ species.

### CO_2_ assimilation rate and carbon isotope discrimination

**Fig. 3.**
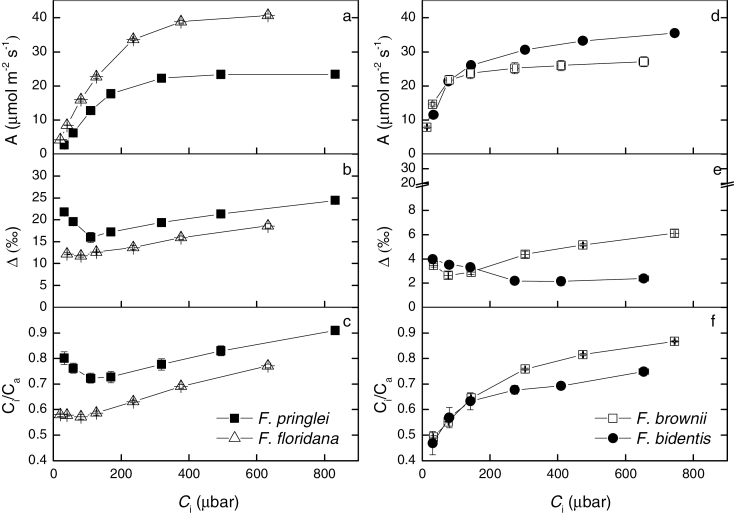
Concurrent measurements of (a, d) CO_2_ assimilation rate, *A*, (b, e) carbon isotope discrimination, *Δ*, and (c, f) the ratio of intercellular to ambient CO_2_, *C*_i_/*C*_a_, as a function of intercellular CO_2_ (*C*_i_) in *F. pringlei*, *F. floridana*, *F. brownii* and *F. bidentis*. Values represent averages and standard error of 4 replicates. Measurements were made at 19 mbar O_2_, a leaf temperature of 25°C and an irradiance of 1500 μmol m^−2^s^−1^.

Measurements of carbon isotope discrimination concurrently with gas exchange were performed under a range of CO_2_ concentrations at 19mbar O_2_ on 3–4 plants from each species (Fig. 3). At this low *p*O_2_, photorespiration is greatly reduced and the effect of the C_2_ cycle is negligible. Thus, small differences in the level of C_4_ activity or mesophyll Rubisco activity are easier to detect.

*F. pringlei* and *F. bidentis* showed the typical C_3_ and C_4_ response of CO_2_ assimilation rate to increasing *C*_i_, respectively (Fig. 3a). The initial slope of the *A*/*C*_i_ curve in *F. floridana* was closer to that in the C_3_ species, *F. pringlei*, whereas that of *F. brownii* was more similar to that of the C_4_ species, *F. bidentis*, although in both intermediate species the sharp saturation typical of the C_4_ species was missing. The maximum apparent assimilation rates in both intermediates were higher than those of the C_3_ and C_4_ species.

Carbon isotope discrimination measured over the defined range of *p*CO_2_ provided clear differences between the four species (Fig. 3b). *Δ* was greatest in *F. pringlei* at any *C*_i_, ranging from 16‰ to 24.4‰. Discrimination in *F. floridana* followed a similar trend than that in the C_3_ species, with *Δ* generally increasing with *C*_i_, but *Δ* was lower than in *F. pringlei* across the whole experimental range, ranging from 12.2‰ to 18.6‰. The response of *C*_i_/*C*_a_ to CO_2_ concentration was parallel to that of *Δ* in *F. pringlei* and *F. floridana*, reflecting the strong dependence of *Δ* on the ratio *C*_i_/*C*_a_ in C_3_ species and also in *F. floridana* (Fig. 3c). The initial decrease of *Δ* in *F. pringlei* is also caused by a drop in *C*_i_/*C*_a_, which is in turn driven by a reduction of stomatal conductance with increasing *C*_i_ when *C*_i_ is lower than 200μbar.

In *F. bidentis*, as expected from a C_4_ plant, discrimination was low (2–4‰) and decreased slightly with increasing *C*_i_. *Δ* in *F. brownii* was similar to *F. bidentis* at *C*_i_ under 95 μbar (3.5–2.6‰), but above that the value of *Δ* increased with increasing *C*_i_, to a maximum of 6.1‰.

**Fig. 4.**
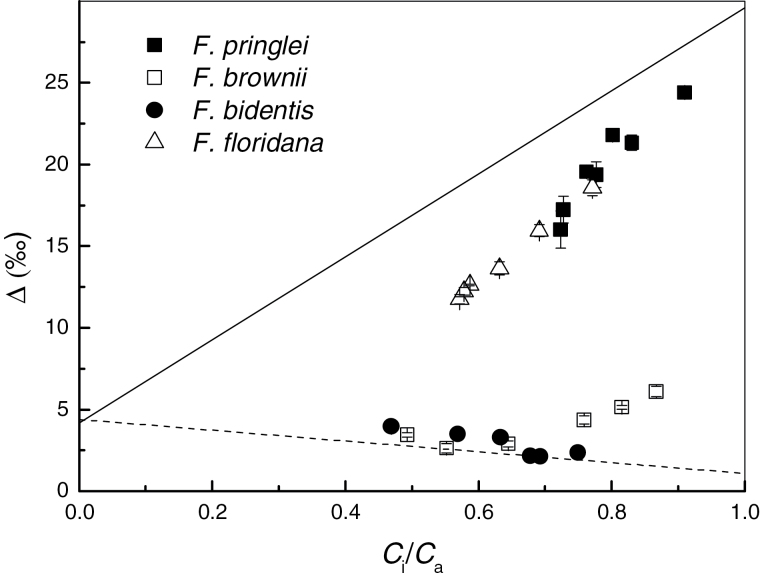
Observed carbon isotope discrimination, *Δ* expressed as a function of the ratio of intercellular to ambient CO_2_, *C*_i_/*C*_a_, in *F. pringlei*, *F. floridana*, *F. brownii* and *F. bidentis*. Values are the same as plotted in Fig. 3. Solid line represents the theoretical response of *Δ* to *C*_i_/*C*_a_ in C_3_ plants ($\Delta =4.4+\frac{C_{i}}{C_{a}}(29-4.4)$ ; (Roeske and O’Leary, 1984; Evans *et al.*, 1994). Dashed line represents the theoretical response of *Δ* to *C*_i_/*C*_a_ in C_4_ plants, $\Delta =4.4+\frac{C_{i}}{C_{a}}[-5.7-4.4+\phi \left(29-1.8\right)]$ (Henderson *et al.*, 1992) when φ=0.25.

Measured *Δ* is shown with respect to *C*_i_/*C*_a_ in Fig. 4. The theoretical lines assume infinite mesophyll conductance, which explains why both *F. pringlei* and *F. floridana* fell below the theoretical response for C_3_ plants, with *Δ* and *C*_i_/*C*_a_ generally lower in *F. floridana*. In *F. bidentis*, the result was as predicted by a theoretical CO_2_ response of *Δ* for a C_4_ plant when ϕ=0.25, whereas *F. brownii* only fitted the expected response at low *C*_i_/*C*_a_, with *Δ* higher than predicted at high *C*_i_/*C*_a_.

### Modeling CO_2_ assimilation rate and carbon isotope discrimination in C_3_-C_4_ intermediate species

**Fig. 5.**
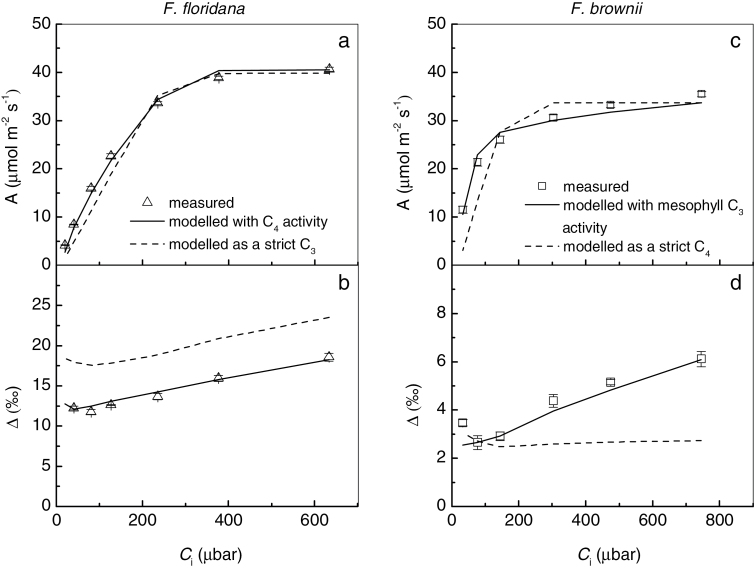
Comparison between modeled and measured responses of CO_2_ assimilation rate, *A*, and carbon isotope discrimination, *Δ*, to variation in intercellular *p*CO_2_, *C*_i_, in the C_3_-C_4_ intermediate species *F. floridana* and *F. brownii*. Measured *A* (a) and *Δ* (b) as a function of *C*_i_ in *F. floridana* (empty triangles), compared with the modeled responses predicted by C_3_-C_4_ photosynthetic model assuming an active C_4_ cycle (solid lines) or no C_4_ cycle activity (dashed lines). Measured *A* (c) and *Δ* (d) as a function of *C*_i_ in *F. brownii* (white squares), compared with the modelled responses using the C_3_-C_4_ models assuming Rubisco activity in the mesophyll cells (solid lines) or a strict compartmentalization of Rubisco in the bundle sheath cells (dashed lines). Parameters used for model simulations are presented in Table 1.

In order to evaluate the contribution of the C_4_ cycle to overall photosynthesis in the intermediate species *F. floridana* and *F. brownii*, the mathematical models proposed here for *A* and *Δ* responses to *C*_i_ (eqs 6 and 10, respectively) were fitted concurrently to the observed results (Fig. 5). By simultaneously fitting both models using the same set of parameters, the accuracy of the predictions increases because some combinations of assigned constants that may result in a good fit for one of the models are unacceptable for the other. For comparison, the same strategy was also applied to the C_3_ and C_4_ species (see Supplementary Fig. S2).

Table 1 shows the values assigned for fitting purposes and their source. Rubisco *K*_C_ and *K*_O_ (Michaelis–Menten constants for CO_2_ and O_2_, respectively) in the four *Flaveria* species analyzed here have been previously reported (Kubien *et al.*, 2008), and *V*_c,max_ and *V*_p,max_ are from our own *in vitro* experiments. We assigned reasonable values for maximum electron transport (*J*_max_). Leakiness(ϕ) was assigned so that the sum of the squares of the variances between the measured and modeled *A*, and between the measured and modeled *Δ*, was minimum. The distribution of Rubisco between the mesophyll and the bundle sheath in the intermediate species can be adjusted in the models by the assigned *V*_m,max_ (maximum rate of Rubisco carboxylation in the mesophyll) value. When *V*_m,max_ equals the *V*_c,max_ observed *in vitro*, all Rubisco is in the mesophyll. A lower asigned *V*_m,max_ indicates that part of the Rubisco activity is contained in the bundle sheath cells.

**Fig. 6.**
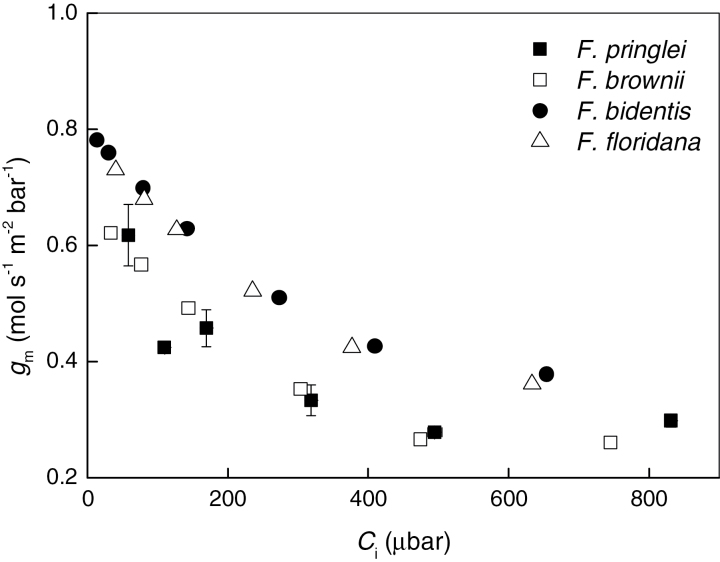
Response of mesophyll conductance (*g*_m_) to changes in atmospheric *p*CO_2_. In *F. pringlei*, *g*_m_ was calculated from concurrent gas exchange and *Δ* measurements made at 19mbar *p*O_2_. The values for *F. floridana*, *F. brownii* and *F. bidentis* were assigned assuming the same response of *g*_m_ to *C*_i_ as observed in *F. pringlei*, scaled from model fitting.

Mesophyll conductance (*g*_m_) for *F. pringlei* was calculated from concurrent gas exchange and carbon isotope discrimination measurements at 19mbar O_2_ and a range of reference *p*CO_2_ as previously described (Tazoe *et al.*, 2011; Farquhar and Cernusak, 2012; Evans and von Caemmerer, 2013). Results show that *g*_m_ decreases from 0.62±0.1 to 0.33±0.03mol m^–2^ s^–1^ bar^–1^ with increasing *C*_i_ when atmospheric *p*CO_2_ is lower than ambient, and then remains stable at higher *p*CO_2_ (Fig. 6). The CO_2_ dependence of *g*_m_ in *F. pringlei* is described by the polinomial function *g*_m_=10^–6^·*C*_i_^2^−0.0013·*C*_i_+*c*, where *c*=0.666. In C_4_ and C_3_-C_4_ intermediate species, *g*_m_ cannot be obtained from concurrent gas exchange and *Δ* ^*13*^C measurements, so the same CO_2_ dependence of *g*_m_ was assumed for *F. bidentis*, *F. brownii* and *F. floridana*, and the constant *c* was calculated from model fitting so that the sum of variances between the measured and modeled *A*, and between the measured and modeled *Δ*, was minimum (Table 1). The resulting *g*_m_ are shown in Fig. 6. Methods for obtaining *g*_m_ in C_4_ and C_3_-C_4_ intermediate species, based on ^18^O discrimination measurements, are currently being developed (S. von Caemmerer, unpublished results).

The *A* and *Δ* responses to increasing *C*_*i*_ predicted with this strategy were reasonably close to the measured values for *F. pringlei* and *F. bidentis* (Supplementary Fig. S2).

In an exercise to prove the predictive value of these models for the presence of low levels of activity of the C_4_ component, we attempted to fit the models for *F. floridana* under two different premises. In one case, we assumed a certain level of effective C_4_ cycle contribution to overall carbon assimilation (Fig. 5a, b, solid lines). In the second case, we considered no C_4_ activity and values were assigned to obtain the best possible fitting ignoring the measured enzyme activities (Fig. 5a, b, dashed lines). The models could only be fitted to the measured values of *Δ* and *A* if some C_4_ activity, specified by a *V*_p,max_ close to our *in vitro* measurements, was assumed.

A similar approach was used with *F. brownii*. In one case, the models were fitted assuming the presence of Rubisco in the mesophyll, and in the other case the model was fitted as if it were a strict C_4_ plant (Fig. 5c, d). The predicted responses approached the measured values only if ~30% of Rubisco activity was located in the mesophyll (*V*_m,max_ =15 μmol m^−2^ s^−1^; observed *in vitro V*_c,max_ =50 μmol m^−2^ s^−1^).

**Fig. 7.**
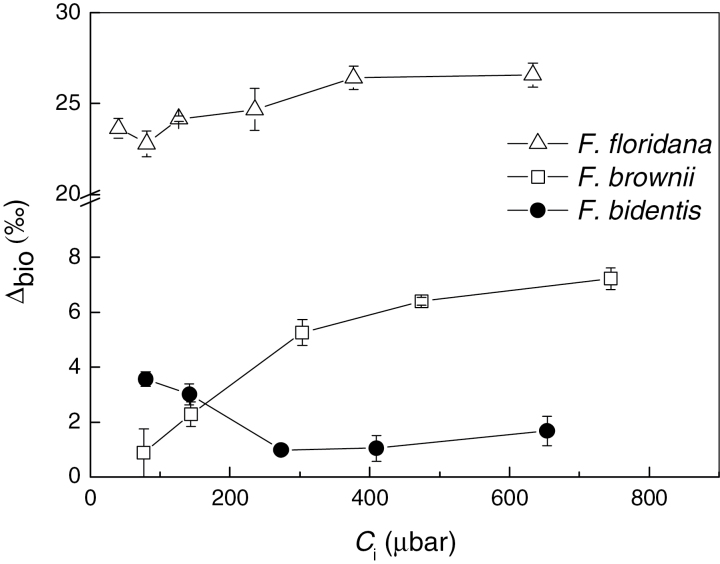
Biochemical fractionation (*Δ*_bio_), as a function of intercellular CO_2_ (*C*_i_) in *F. floridana*, *F. brownii* and *F. bidentis*. *Δ*_bio_ was calculated from eq. 12 using the combined gas exchange and *Δ* measurements shown in Fig. 3. *Δ*_bio_ could not be calculated for *F. pringlei* because *g*_m_ is obtained from *Δ* measurements in this species, so both factors are not independent. Values represent averages and standard error of four replicates.

A comparison of *Δ* and *Δ*_bio_ highlights the fact that CO_2_ diffusion processes have a large influence on *Δ* (Figs 3, 7). *Δ*_bio_ was calculated from eq. 12 using gas exchange and *Δ* measured values. Calculation of *Δ*_bio_ factors out the contribution from CO_2_ diffusion and shows that the biochemical fractionations are different in the species analyzed. In *F. floridana*, *Δ*_bio_ was high and increasing with *C*_i_. In *F. brownii*, *Δ*_bio_ also increased with increasing *C*_i_, whereas in the C_4_ species *F. bidentis Δ*_bio_ generally decreases with *C*_i_.

The *A* and *Δ* responses to *C*_i_ could be modeled assuming a constant *g*_m_ without important differences (data not shown). However, the calculation of biochemical fractionation (*Δ*_bio_) from eq. 12 is dependent on *g*_m_, and thus the dependence of *g*_m_ on *C*_i_ must have an effect on *Δ*_bio_. To show the magnitude of this effect, the *C*_i_ response of *Δ*_bio_ was calculated from eq. 12 and the gas exchange and *Δ* measurements, assuming either variable *g*_m_, assigned as previously explained in this section, or constant *g*_m_, calculated as the average of the variable *g*_m_ values obtained for each species (see Supplementary Fig. S3). As a reference, the *C*_i_ response of *Δ*_bio_ was calculated from eq. 14 (modelled *Δ*_bio_) after fitting the models for the *C*_i_ responses of *A* and *Δ* using variable *g*_m_.

### Estimation of the C_4_ (bundle sheath) photosynthesis contribution to total photosynthesis

**Fig. 8.**
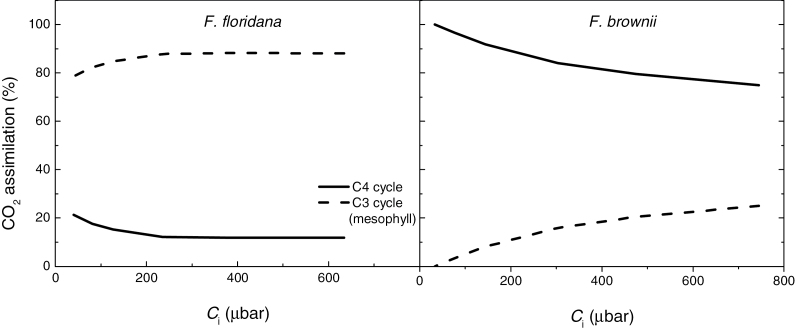
CO_2_ response of the estimated contribution of the C_4_ cycle and the mesophyll C_3_ cycle in the intermediate species *F. floridana* and *F. brownii*, expressed as a percent of total CO_2_ assimilation rate, under low *p*O_2_.

The relative contribution of the bundle sheath to total photosynthesis in the intermediate species was estimated from *A*_s_ in eq. 2, after fitting the models to our observed results (Fig. 8). Because the experiments were performed under low O_2_, photorespiration is greatly reduced and it can be assumed that all the CO_2_ assimilated in the bundle sheath is transported by the C_4_ cycle. The contribution of the bundle sheath to total photosynthesis in both *F. floridana* and *F. brownii* decreased with increasing *C*_i_. In *F. brownii*, almost all carbon was fixed by Rubisco in the bundle sheath at very low *C*_i_, but up to 25% of fixation occurred via Rubisco in the mesophyll at high *C*_i_. In *F. floridana*, the maximum estimated contribution of the bundle sheath photosynthesis via the C_4_ cycle was 21% at very low *C*_i_ and it dropped to 12% at the highest *C*_i_ analyzed.

## Discussion

### Effect of O_2_ on carbon assimilation and compensation point

The oxygen responses of CO_2_ assimilation and the compensation point have been used in the past as a tool to identify and characterize C_3_-C_4_ intermediate species (Sayre and Kennedy, 1977; Monson *et al.*, 1984; Dai *et al.*, 1996; Vogan *et al.*, 2007). As only mesophyll Rubisco is exposed to air oxygen, its effect on CO_2_ assimilation and Γ decreases with increasing proportions of the enzyme allocated to the bundle sheath. However, it is difficult to separate and quantify the effects of the C_2_ and C_4_ cycles from studies on the O_2_ response of CO_2_ assimilation, as both cycles contribute to reduce the negative effect of photorespiration in carbon assimilation and the compensation point. Moreover, the efficiency of the C_2_ cycle varies between different intermediate species, as does the contribution of the C_4_ cycle (Cheng *et al.*, 1988; Keerberg *et al.*, 2014).

In this work, the O_2_ response of carbon assimilation, and especially Γ, in *F. brownii* was very close to that of the C_4_ species *F. bidentis*. A highly efficient C_2_ cycle would have a greater impact on the O_2_ sensitivity of Γ than on carbon assimilation and that, combined with high *in vitro* PEPC and CA activities at the same level as the C_4_ species *F. bidentis*, eliminates the effect of *p*O_2_ on Γ almost completely (Cheng *et al.*, 1988; Ku *et al.*, 1991). Previous studies initially classified *F. brownii* as a C_4_ species, but it was later demonstrated that the enzyme compartmentation is incomplete in this plant (Monson *et al.*, 1987; Ku *et al.*, 1991). The small proportion of Rubisco present in the mesophyll is reflected in the sensitivity of assimilation rate to *p*O_2_.

CA activity in *F. floridana* is similar to *F. pringlei* but PEPC activity is four times higher (13.8 μmol m^−2^ s^−1^), consistent with Ku *et al.* (1991) and supporting the hypothesis of an active C_4_ cycle. However, PEPC activity is still low when compared with *F. bidentis* (91.8 μmol m^−2^ s^−1^), indicating that the activity of the C_4_ cycle in this plant is small. In our experiments, the O_2_ sensitivity of Γ in *F. floridana* is intermediate, and the O_2_ response of CO_2_ assimilation rate is remarkably close to that of *F. brownii*.

Previous studies have reported a C_3_-like O_2_ response in *F. floridana* (Dai *et al.*, 1996; Monson *et al.*, 1986), which differs from our observations. Although the reason for this discrepancy is not known, it must be noted that O_2_ sensitivity measurements are affected by variation of parameters like temperature or stomatal conductance between measurements at different *p*O_2_. These interactions increase the difficulty of estimating the activity of the C_4_ cycle from O_2_ response experiments.

### Signature of C_4_ photosynthesis in the CO_2_ response of *Δ* in intermediate species

The different CO_2_ responses of *Δ* in the intermediate C_3_-C_4_ species, relative to the C_3_ or C_4_ species, can be attributed to the different ratios of PEPC/Rubisco activity in the mesophyll. The lower *Δ* observed in *F. floridana*, relative to *F. pringlei,* is partially attributable to a lower *C*_i_/*C*_a_, but their different *Δ*_bio_ indicates an influence of the PEPC to Rubisco ratio, especially at low *C*_i_.

Interestingly, *F. brownii* and *F. bidentis* show similar *Δ* at low *C*_i_, but it increases in *F. brownii* with increasing *p*CO_2_ instead of decreasing as in the C_4_ plant. This particular response can be attributed to the activity of the small fraction of Rubisco in the mesophyll that would have a stronger influence at high *p*CO_2_. In *F. floridana*, Rubisco is abundant in the mesophyll but PEPC activity is low, and as a consequence the greatest effect of the C_4_ cycle activity is observed at very low *p*CO_2_, with a greater reduction of *Δ* compared to the C_3_ species. Both results indicate that the contribution of mesophyll Rubisco to overall assimilation is more important under high *p*CO_2_, and of the C_4_ cycle at low *p*CO_2_. The fact that environmental conditions affect the contribution of C_4_ photosynthesis may explain ambiguous results on previous analyses of dry matter δ^13^C in *F. floridana* and other intermediates, which showed C_3_-like ratios (Monson *et al.*, 1988; Byrd *et al.*, 1992). δ^13^C is a result of carbon discrimination during the leaf growth, thus it integrates the effect of variable environmental conditions. In the online experiments presented here, instant discrimination is measured under controlled conditions, highlighting their influence. By performing the analyses under low *p*O_2_, the effect of photorespiration and subsequent refixation through the C_2_ cycle is greatly reduced, emphasizing the differences in biochemical fractionation caused by the presence of C_4_ activity.

Although the CO_2_ response of *A* is also influenced by different relative activities of mesophyll Rubisco and PEPC, the effect of each enzyme in this case is difficult to separate. The greater initial slope of the *A*/*C*_i_ curve in *F. floridana*, compared with *F. pringlei*, reflects the slightly greater PEPC activity detected in our *in vitro* assays, but could also be attributed to higher Rubisco activity. In the same sense, the initial slope of the *A*/*C*_i_ curve in *F. brownii* and *F. bidentis* are similar and typically C_4_, whereas their *Δ* are different.

### Concurrent model fitting reveals C_4_ activity in *F. floridana*

The strategy to evaluate the contribution of the C_4_ cycle to total carbon assimilation in intermediate species presented in this work is based on concurrently measuring and model-fitting the CO_2_ responses of carbon assimilation and discrimination.

Mathematical modeling has proved to be a powerful tool to get a deeper insight into the biochemical and physiological basis of the observed responses of carbon assimilation and discrimination, and it has been used to estimate parameters such as the maximum carboxylase activity of Rubisco *in vivo* (*V*_C,max_) and *g*_m_ in C_3_ species, or *V*_P,max_ and leakiness in C_4_ systems (Tazoe *et al.*, 2011; Ubierna *et al.*, 2011; Walker *et al.*, 2013; Sharwood and Whitney, 2014). However, in most cases there is more than one unknown variable in the equations that represent those responses. This is especially problematic in intermediate species, where the number of factors affecting those responses is greater than in C_3_ or C_4_ plants. By concurrently fitting the CO_2_ responses of *A* and *Δ* in each experiment with the same set of constants, the range of values that can be assigned to these variables to obtain a satisfactory fitting is reduced. In this work, the activities of photosynthetic enzymes were analyzed *in vitro* to further reduce the number of unknowns, providing more accurate predictions. This method confirmed the presence of Rubisco activity in the mesophyll of *F. brownii*, which was already known (Cheng *et al.*, 1988), but more interestingly indicated that *F. floridana* harbors an active C_4_ cycle. This C_4_ activity causes a change in the biochemical fractionation, compared to *F. pringlei*, which is evident at any *C*_i_ analyzed. This is consistent with the increased activity of PEPC and previous observations based on ^14^CO_2_ pulse-chase experiments (Monson *et al.*, 1986; von Caemmerer and Hubick, 1989). It is important to note that other studies based on δ^13^C analyses, metabolite dynamics and O_2_ response of carbon assimilation and Γ were unable to conclusively prove a contribution of the C_4_ cycle to overall photosynthesis in *F. floridana*, and the presence of a futile C_4_ cycle was proposed where most or all the CO_2_ released in the bundle sheath is not fixed and leaks back to the mesophyll (Monson *et al.*, 1988; Leegood and von Caemmerer, 1994; Dai *et al.*, 1996). However, other authors have already indicated that in *F. floridana* the C_4_ cycle may contribute up to 50% of the total CO_2_ fixation (Ku *et al.*, 1991). In this work, the contribution of the mesophyll and the bundle sheath Rubisco to overall carbon assimilation was calculated for *F. brownii* and *F. floridana*. In both intermediate species, the contribution of the C_4_ cycle, or bundle sheath Rubisco, is highest at very low *p*CO_2_, and decreases with increasing *p*CO_2_. This reflects the lower apparent *K*_c_ of PEPC compared to that of Rubisco (Bauwe, 1986; Kubien *et al.*, 2008).

### An improved equation describing CO_2_ response of *Δ* in intermediate species

An equation describing photosynthetic carbon isotope discrimination (*Δ*) that is applicable for C_3_, C_4_ and C_3_-C_4_ photosynthesis is provided and applied in this study. It allows the calculation of the biochemical fractionation occurring for the different photosynthetic pathways as a function of *C*_i_ and takes into account *g*_m_ and the ternary effects of transpiration rate. The biological relevance of *g*_m_, and its influence on *Δ*, has been reported extensively and incorporated in mathematical models for C_3_ species (Evans *et al.*, 1986; von Caemmerer and Evans, 1991; Tazoe *et al.*, 2011). When mesophyll conductance is considered in C_3_ species, *C*_c_ (*p*CO_2_ at the site of Rubisco) can be estimated and is lower than *C*_i_, and this affects the estimates of Rubisco carboxylations. The same applies in intermediate species, where assimilation and discrimination by mesophyll Rubisco is dependent on the concentration of CO_2_ diffusing from the intercellular space. For model fitting purposes, the calculated *C*_c_ was used as the available CO_2_ for both PEPC and mesophyll Rubisco in the case of the intermediate species. The models presented in this work assume that *p*CO_2_ is the same in the cytosol and the chloroplast.

The effect of *p*CO_2_ on *g*_m_ has been studied by other authors, with results depending on the species analyzed. Whereas previous results showed that *g*_m_ is not affected by *p*CO_2_ in wheat (Tazoe *et al.*, 2009), other authors reported an inverse correlation in several C_3_ species (Flexas *et al.*, 2007; Tazoe *et al.*, 2011). We observed that *g*_m_ is dependent on *p*CO_2_ in the C_3_ *F. pringlei*, and assumed that the same is true for the C_4_ and intermediate species analyzed. Although the effect of using either constant or variable *g*_m_ on the models of the CO_2_ responses of carbon assimilation and discrimination has only a minor effect at low *C*_i_, it is important for the calculation of *Δ*_bio_ and thus the contribution of the C_4_ and C_3_ cycles to overall carbon assimilation, especially at low *C*_i_. The fact that *Δ*_bio_ is similar when calculated using either constant or variable *g*_m_ in *F. brownii* and *F. bidentis* reflects the lower relevance of *g*_m_ when the CO_2_ concentrating mechanism is expressed at high levels.

## Conclusion

Concurrent *Δ* and gas exchange measurements and modeling provide a powerful diagnostic tool for C_4_ photosynthesis. Performing the measurements under controlled environmental conditions, especially low *p*O_2_, allows the detection and estimation of the C_4_ cycle activity in C_3_-C_4_ intermediate species even when it is low. This approach confirmed the presence of active Rubisco in the mesophyll of *F. brownii*, and revealed a contribution of the C_4_ cycle to total carbon assimilation in *F. floridana*. However, the carbon isotope signal is complex and not all its components are well understood, so some caution is required. We show for example that a CO_2_ dependence of g_m_ affects the calculation of the biochemical fractionation, and thus the contribution of the C_4_ cycle to overall CO_2_ assimilation.

## Supplementary data

Supplementary data are available from *JXB* online.

Figure S1. Responses of *C*_i_ and stomatal conductance to changes in atmospheric *p*O_2_.

Figure S2. Models of CO_2_ response of assimilation rate and carbon isotope discrimination in the C_3_ and C_4_ species.

Figure S3. Effect of assuming constant or variable *g*_m_ in the calculation of the biochemical fractionation.

Supplementary Data
