# Supplementary material for: Carbon isotope discrimination as a diagnostic tool for C4 photosynthesis in C3-C4 intermediate species
Source: J Exp Bot. 2016 Feb 8;67(10):3109–21. doi: 10.1093/jxb/erv555 (PMC4867892; doi:10.1093/jxb/erv555)
Supplement: Supplementary Data [file supp_67_10_3109__index.html]

Carbon isotope discrimination as a diagnostic tool for C4 photosynthesis in C3-C4 intermediate species — Carbon isotope discrimination as a diagnostic tool for C4 photosynthesis in C3-C4 intermediate species — Supplementary Data 

# Carbon isotope discrimination as a diagnostic tool for C4 photosynthesis in C3-C4 intermediate species

## Supplementary Data

Data files

- supplementary\_figures\_S1\_S3.pdf - Supplementary Data
