# Supplementary material for: Carbon isotope discrimination as a diagnostic tool for C4 photosynthesis in C3-C4 intermediate species
Source: J Exp Bot. 2016 Feb 8;67(10):3109–21. doi: 10.1093/jxb/erv555 (PMC4867892; doi:10.1093/jxb/erv555)
Supplement: Supplementary Data [file supp_erv555_supplementary_figures_S1_S3.pdf]

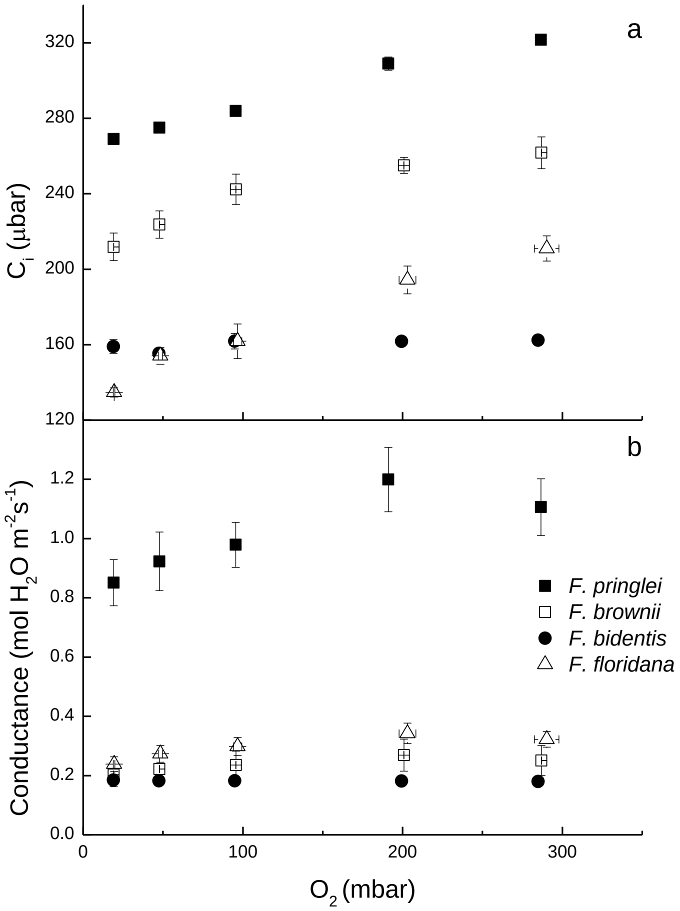

Figure S1.

The responses of C_i (**a**) and stomatal conductance (**b**) in *F. pringlei*, *F. floridana*, *F. brownii* and *F. bidentis* to changes in atmospheric pO_2. Results correspond to the same measurements as Fig. 1a. Values represent averages and standard error of 4 replicates.

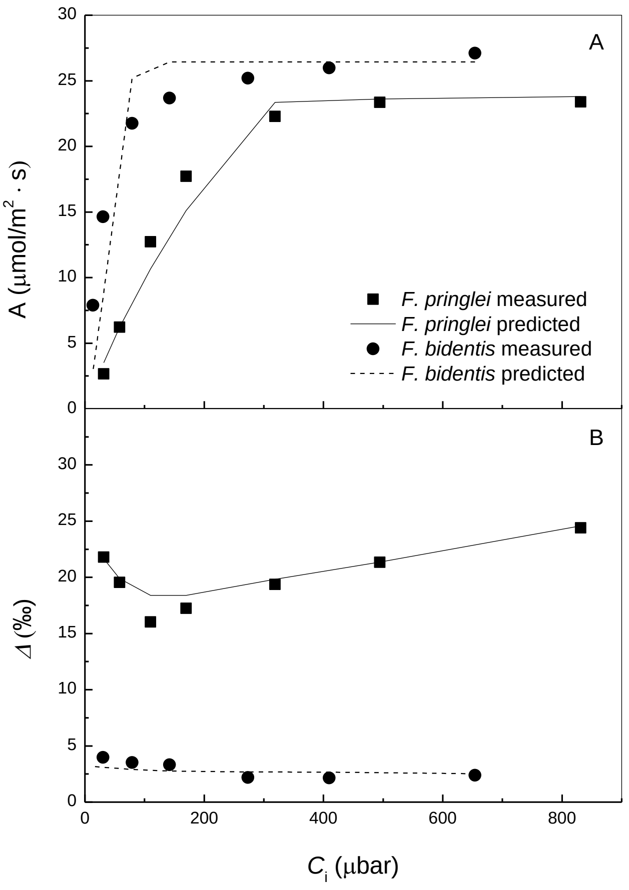

Figure S2.

Models of CO_2 response of assimilation rate and carbon isotope discrimination in the C_3 and C_4 species. (**a**) Measured CO_2 response of *A* in *F. pringlei* and *F. bidentis*, compared with the predicted responses. (**b**) Measured CO_2 response of Δ in *F. pringlei* and *F. bidentis*, compared with the predicted responses. Parameters used for modelling are presented in Table 1.

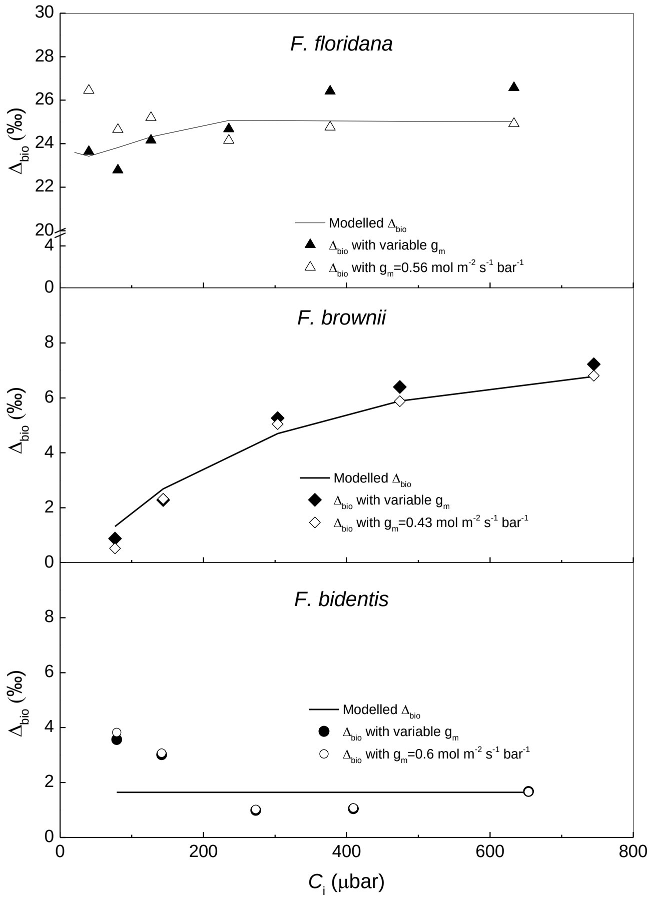

Figure S3.

The effect of assuming constant or variable g_m in the calculation of the biochemical fractionation (Δ_{bio}) in *F. floridana*, *F. brownii* and *F. bidentis*. Δ_{bio} was calculated from eq. 12 using gas exchange and Δ measurements, and assuming constant (open symbols) or variable g_m (solid symbols). The variable g_m values applied are shown in Fig. 6. Constant g_m is the average of the variable values obtained for each species. Modelled Δ_{bio} (solid lines) was calculated from eq. 14 using the photosynthesis model with the parameters given in Table 1. F. *bidentis* is considered a strict C_4, thus modelled Δ_{bio} is constant and independent from C_i and g_m.
